# Early operative morbidity in 184 cases of anterior vertebral body tethering

**DOI:** 10.1038/s41598-021-02358-0

**Published:** 2021-11-29

**Authors:** James Meyers, Lily Eaker, Theodor Di Pauli von Treuheim, Sergei Dolgovpolov, Baron Lonner

**Affiliations:** 1 Icahn School of Medicine at Mount Sinai, New York, NY USA; 2 Department of Orthopedic Surgery, Icahn School of Medicine, Mount Sinai Hospital, New York, NY USA; 3 Department of Surgery, Mount Sinai Hospital, New York, NY USA

**Keywords:** Outcomes research, Paediatric research

## Abstract

Fusion is the current standard of care for AIS. Anterior vertebral body tethering (AVBT) is a motion-sparing alternative gaining interest. As a novel procedure, there is a paucity of literature on safety. Here, we report 90-day complication rates in 184 patients who underwent AVBT by a single surgeon. Patients were retrospectively reviewed. Approaches included 71 thoracic, 45 thoracolumbar, 68 double. Major complications were those requiring readmittance or reoperation, prolonged use of invasive materials such as chest tubes, or resulted in spinal cord or nerve root injury. Minor complications resolved without invasive intervention. Mean operative time and blood loss were 186.5 ± 60.3 min and 167.2 ± 105.0 ml, respectively. No patient required allogenic blood transfusion. 6 patients experienced major (3.3%), and 6 had minor complications (3.3%). Major complications included 3 chylothoracies, 2 hemothoracies, and 1 lumbar radiculopathy secondary to screw placement requiring re-operation. Minor complications included 1 patient with respiratory distress requiring supplementary oxygen, 1 superficial wound infection, 2 cases of prolonged nausea, and 1 Raynaud phenomenon. In 184 patients who underwent AVBT for AIS, major and minor complication rates were both 3.3%.

## Introduction

Adolescent Idiopathic Scoliosis (AIS) is a structural disease of the spine resulting in abnormal lateral curvature and rotation. It presents in approximately 3% of adolescents who develop spinal curvature > 10°, making it the most common etiology of scoliosis. AIS is associated with a number of detrimental consequences including increased back pain^1^, decreased range of motion^2^, psychological distress^3,4^, and impaired pulmonary function in severe cases^5–7^.

The standard surgical treatment for AIS is spinal fusion, first performed over a century ago. Since then, there have been multiple paradigm shifts in surgical technique^8,9^, from the introduction of spinal instrumentation in the 1950s, anterior instrumentation in the 1970’s, and pedicle screws in the 1990s. The last 20 years has seen a steady decline of anterior spinal fusion (ASF) and a reciprocal increase in posterior spinal fusion (PSF)^9^ seemingly due to the balance of efficacy, decreased complications, and better pulmonary outcomes relative to open anterior approaches^10–13^. Minimally invasive anterior alternatives include an anterior thoracoscopic approach and a thoracoscopically-assisted-mini-open approach^14^. The use of thoracoscopic procedures, however, is infrequent in part due to a steep learning curve^15,16^ and greater incidence of complications^15,17,18^. Thus, the current surgical gold standard remains PSF^19^.

PSF is not without its limitations including compromised mobility within the instrumented segments and the potential for decreased overall flexibility, adjacent disc disease^20^, and flatback syndrome^21–24^. PSF may also be less effective than ASF at remedying hypokyphosis and restoring sagittal contour^25^. It also requires more muscle dissection and leads to more post-operative pain than the anterior thoracoscopic approach^15^.

Anterior Vertebral Body Tethering (AVBT) is a motion-sparing alternative that has been introduced with some success^26,27^. It uses an anterior thoracoscopically-assisted approach and may avoid some of the drawbacks associated with PSF. However, as a novel procedure, there is a paucity of literature on safety. Here, in the largest consecutive series to date, we report and characterize the 90-day complication rates in 184 patients who underwent AVBT by a single surgeon.

## Materials and methods

All methods were carried out in accordance with relevant guidelines and regulations.

### Patients

All patients were counseled on their surgery options and concomitant risks and benefits, as well as what remains unknown regarding AVBT including timing of surgery in relation to peak height velocity, and uncertain variables such as time until possible cord breakage. More skeletally mature patients who were offered AVBT were counseled about the lack of data for skeletally mature patients and the potential for future revision surgery, including PSF. Only after it was determined that the patient’s priorities and expectations aligned with the potential advantages and drawbacks of AVBT was the surgery deemed appropriate. General indications included diagnosis of AIS, no prior spinal surgery, and no chronic co-morbidities.

### Chart review

Approval for a retrospective chart review and waiver of informed consent was obtained from the Mount Sinai School of Medicine Institutional Review Board (IRB). 184 consecutive AVBT cases, performed by a single surgeon, were included. Patient medical records were reviewed for the following demographic and surgical characteristics: age, sex, estimated blood loss (EBL), rib resection, pre-operative Cobb angle, operative time, tether location, number of tether cords, number of levels tethered, and complications. Rib resection was analyzed as a yes/no variable, inclusive of individual ribs removed (1or 2) to facilitate screw placement and also multiple (3+) ribs (thoracoplasty) removed to address severe rib deformity. Data were collected by two trained research associates and validated by an attending orthopedic spine surgeon.

### Complications

Complications were divided into eight domains consistent with previous studies: Pulmonary, Neurological, Gastrointestinal, Cardiovascular, Instrumentation, Pain, Wound/Infection, and Other^28–30^. Medical records in an EPIC Hyperspace database were analyzed for complications. Searchable keywords were identified for each complication domain and were used as a post-hoc verification tool. After complications were identified, they were further divided by time and severity. For descriptive purposes, perioperative complications were defined as occurring within 90 days of surgery. Major complications were defined as those that required readmittance or reoperation, prolonged the use of invasive materials such as chest tubes, or caused injury to a nerve root or the spinal cord. Minor complications were those that resolved without invasive intervention.

### Statistical analyses

For continuous variables, normality was evaluated with a Kolmogorov–Smirnov test. Parametric tests were used where distributions were normal and non-parametric tests were used when distributions were not normal (*p* < 0.05). First, demographic and clinical features were compared among patients who experienced any complications and those who did not, and then between patients who experienced major complications and those who did not (Table 1). A binomial logistic model was created with pre-operative Cobb angle, rib resection, number of levels tethered, and whether one or two curves were instrumented as independent variables, while complication outcome (dichotomous yes/no) was the dependent variable. This was done twice to produce an *all-complication* model and a *major-complication* model. These analyses generated odds ratios (OR) with 95% confidence intervals (CI). Analyses were executed with SPSS software, IBM Corp., Armonk, NY.

**Table 1:** Demographic and clinical features divided by complications.

Demographic and clinical features	All patients N = 184	All complications N = 12	No complications N = 172	Sig	Major complications N = 6	No Major complications N = 178	Sig
Age	15.0 ± 2.4	14.8 ± 2.7	15.1 ± 2.4	*p* = 0.77a	15.5 ± 3.6	15.0 ± 2.3	*p* = 0.64a
Female Sex	143 (77%)	11 (7.7%)	132 ± (92.3%)	*p* = 0.31c	5 (3.5%)	138 (96.5%)	*p* = 0.74c
EBL (ml)	167.2 ± 105.0	193.8 ± 94.2	165.4 ± 105.8	*p* = 0.20b	200.0 ± 83.7	166.1 ± 105.7	*p* = 0.22b
%EBV	4.4% ± 2.9%	5.6% ± 2.4%	4.3% ± 2.9%	***p* = 0.04b**	5.3% ± 2.2%	4.4% ± 2.9%	*p* = 0.20b
Pre-Op Cobb	54.4 ± 10.5	51.8 ± 10.3	54.5 ± 10.6	*p* = 0.48b	51 ± 12.7	54.4 ± 10.5	*p* = 0.50b
Operative time	186.5 ± 60.3	190.9 ± 67.3	186.2 ± 60.0	*p* = 0.82b	179 ± 56.4	186.7 ± 60.6	*p* = 0.82b
Levels tethered	8.1 ± 2.0	8.2 ± 2.2	8.1 ± 2.0	*p* = 0.99b	7.8 ± 2.2	8.1 ± 2.0	*p* = 0.80b
Single-corded tether	121 (65.8%)	9 (7.4%)	112 (92.6%)	*p* = 0.75d	3 (2.5%)	118 (97.5%)	*p* = 0.41d
Two tethered curves	68 (37.0%)	9 (13.2%)	59 (86.8%)	***p* = 0.01d**	4 (5.9%)	64 (94.1%)	*p* = 0.20d
Thoracic only	71 (38.6%)	1 (1.4%)	70 (98.6%)	***p* = 0.03d**	1 (1.4%)	70 (98.6%)	*p* = 0.41d
Thoracolumbar only	45 (24.5%)	2 (4.4%)	43 (95.6%)	*p* = 0.73d	1 (2.2%)	44 (97.8%)	*p* > 0.99d
Rib resection	22 (12.0%)	4 (18.2%)	18 (81.8%)	***p* = 0.04d**	3 (13.6%)	19 (86.4%)	***p* = 0.02d**
No Rib resection	162 (88%)	8 (4.9%)	154 (95.1%)	***p* = 0.04d**	3 (1.9%)	159 (98.1%)	***p* = 0.02d**

Significant values are in [bold].

a = Student’s T-test.

b = Mann–Whitney.

c = Chi Square.

d = Fisher's Exact.

e = Kruskal-Wallace.

## Results

### Demographic and clinical features

Of 184 consecutive AVBT cases, 143 (77.7%) were female and 41 (22.3%) were male. The mean age at surgery was 15.0 ± 2.4 years and differed between males (16.1 ± 1.9) and females (14.7 ± 2.4, *p* = 0.001). The mean pre-operative Cobb angle was 54.3° ± 10.5° and was similar between genders (*p* = 0.06). There were 22 (12.0%) patients who had a rib resection and the mean number of ribs resected was 2.5 ± 1.2. Mean operative time was 186.5 ± 60.3 min and mean EBL was 167.2 ± 105.0 ml. No patient received an allogenic blood transfusion. Patients received either a thoracic tether (71 [38.6%]), thoracolumbar tether (45 [24.5%]), or both (68 [37.0%]) and of these, 121 (65.8%) had single-corded tethers while 63 (34.2%) had double-corded tethers on at least one curve.

### All complications

There were 12 complications in 12 patients (6.5%) within 90 days of surgery. Six patients experienced major complications (3.3%): 3 chylothoraces (1 R Thoracic, 1 L Thoracolumbar, and 1 R Thoracic and L Thoracolumbar, with left-sided chylothorax), 2 hemothoraces (2 R Thoracic and L Thoracolumbar), and 1 lumbar radiculopathy secondary to screw impingement in the neural foramen requiring re-operation. Additionally, 6 patients experienced minor complications (3.3%): 2 patients with oxygen desaturation requiring supplementary oxygen, 1 superficial wound infection, 2 cases of prolonged nausea, and 1 Raynaud phenomenon in both upper and lower extremities ipsilateral to the approach side (Table 2).

**Table 2:** Complication types.

90-Day complications in 184 AVBT Cases	Total complications N = 12	Major complications N = 6	Tethers on two curves N = 9	Rib resection N = 4
Pulmonary	7 (3.8%)	5 (2.7%)	5 (2.7%)	4 (2.2%)
Chylothorax	3 (1.6%)	3 (1.6%)	1 (0.54%)	2 (1.1%)
Hemothorax	2 (1.1%)	2 (1.1%)	2 (1.1%)	1 (0.54%)
Pulm. Insufficiency	2 (1.1%)	0 (0.0%)	2 (1.1%)	1 (0.54%)
Instrumentation	1 (0.54%)	1 (0.54%)	1 (0.54%)	0 (0.0%)
Prolonged Nausea	2 (1.1%)	0 (0.0%)	2 (1.1%)	0 (0.0%)
Wound/Infection	1 (0.54%)	0 (0.0%)	1 (0.54%)	0 (0.0%)
Other	1 (0.54%)	0 (0.0%)	0 (0.0%)	0 (0.0%)

There were 22 patients (12.0%) who received a rib resection, and this was associated with a greater total complication rate, (4/22 = 18.2%), than patients who did not receive a rib resection (8/162 = 4.9%, *p* = 0.04, Table 1); these complications included 2 chylothoraces, 1 hemothorax, and 1 pulmonary insufficiency (Table 2). When parsed by tether location, a greater proportion of complications occurred in patients with 2 curves tethered (9/68 = 13.2%), as opposed to either only thoracic (1/71 = 2.2%) or only thoracolumbar (2/45 = 4.4%) curves (*p* = 0.01, Table 1). These complications included 2 hemothoracies, 2 pulmonary insufficiency, 2 prolonged nausea, 1 chylothorax, 1 superficial wound infection, and 1 instrumentation (Table 2).

In the all-complication binomial logistic model, tether location and rib resection were both associated with complications (*p* = 0.001 and *p* = 0.037 respectively). The model was statistically significant, χ^2^(4) = 15.9, *p* = 0.003, and correctly classified 92.4% of cases. Patients who received a rib resection were 4.6 times more likely to experience a complication than those who did not (95% CI 1.10–19.13). Patients with tethers on both thoracic and thoracolumbar curves were 13.2 times more likely to have a complication (95% CI 2.70–64.45) than patients who received a tether on only one curve. The number of levels tethered, and pre-operative Cobb angle were not significantly associated with complications (*p* = 0.082 and *p* = 0.321 respectively).

### Major complications

Major complications were predominately pulmonary (5 [2.7%]), with 1 (0.54%) instrumentation (Table 2). Rib resection was associated with a greater major complication rate, (3/22 = 13.6%; 2 chylothoraces, 1 hemothorax), than cases with no rib resection (3/162 = 1.9%, *p* = 0.02, Table 1). In the major-complication binomial logistic model patients with a rib resection were 9.4 times more likely to have a major complication (95% CI 1.60–55.53, *p* = 0.013). The model was statistically significant, χ^2^(4) = 9.7, *p* = 0.047, and correctly classified 96.7% of cases. Unlike the all-complication model, tether location was not a significant predictor for major complications (OR 6.59, 95% CI 0.80–53.96, *p* = 0.079).

## Discussion

In the present study, we report the 90-day post-operative complications in 184 consecutive cases of AVBT. Rib resection and two-instrumented curves appear to predict the all-complication rate, but only rib resection was significant for major complications. The all-complication rate was 6.5% and was evenly split between major (3.3%) and minor (3.3%) complications. The major complications were predominately pulmonary in nature (5/6 = 66.7%) with 3 chylothoracies and 2 hemothoracies.

Currently, PSF is the standard of care for patients with AIS, but alternatives such as AVBT have been introduced to sidestep some of the drawbacks of fusion. In order for stakeholders to appropriately balance their needs and concerns when opting for surgery, they must understand the costs, benefits, and risk profiles of those options. Reported benefits of anterior thoracoscopic approaches over fusion include less blood loss^17,18^, less muscle dissection^31^, less post-operative pain^32^, fewer levels instrumented^33^, and improved self-image and mental health SRS-22 scores compared to standard open thoracotomy approaches^34^. However, those same studies reported drawbacks including increased operative time, a steep learning curve, and increased incidence of pulmonary complications. Moreover, the long-term risks with AVBT are unknown, however cord breakage and revision have been reported^35,36^.

Without a PSF control group, we cannot directly compare our AVBT cohort to PSF, however it is worth noting that the complication profiles appear to be different. The proportion of major pulmonary complications reported here (5/6 = 83.3%) is greater than that historically reported for idiopathic scoliosis patients treated with PSF. For example, two large studies that addressed PSF in idiopathic scoliosis, Bartley et al.and Reames et al., reported perioperative pulmonary complication rates of 13/93 (14.0%) and 68/710 (9.6%) respectively^30,37^. Those same studies found that for PSF, neurological deficits were the most common complication [19/93 (20.4%) and 139/710 (19.6%) respectively], followed by instrumentation [15/93 (16.1%) and 120/710 (16.9%) respectively]. This accords with previous studies that reported thoracoscopic anterior approaches are associated with a greater incidence of pulmonary complications relative to posterior approaches^11–13,17^, and may be an important factor when deciding between various surgical options.

Chylothoracies made up a disproportionate amount of the major complications [3/6 (50%)] reported here. Two of the three cases occurred in patients in whom double cords were placed on one side of the spine and in one patient a disc release was performed. The common thread is that more extensive dissection for double-corded tethers or disc release may have led to the inadvertent injury of the thoracic duct or tributaries thereof. The leaks occurred in both right and left sided approaches suggesting no predilection for one side or another. Avoidance of such injuries should be aided by vigilant adherence to standard surgical principles, i.e., dissection of tissues only with full visualization and retraction, and blunt dissection of structures anterior to the spine away from where the sharp dissection is performed which should be over the spine directly.

This study suffers from several limitations. Our sample sizes are too small to permit multivariate analysis of factors associated with complications. Future studies should continue to add to the data and parse out the types of complications seen, as they may differ in character and evolve as surgeons become more comfortable with AVBT. Nonetheless, it is noteworthy that even with a small sample size, a pulmonary theme emerged. Additionally, we were not able to statistically compare PSF to AVBT in terms of complication rates or unique complication profiles, but as data becomes available researchers should pursue those goals.

Finally, we have included in this cohort, patients who have been treated outside of the standard indications for AVBT based on skeletal maturity. There are 18 patients between the ages of 18–21 who have surpassed peak growth velocity and are the subject of ongoing study. Skeletal maturity implies little ability for AVBT to modulate growth and means that curve correction will be maintained by the implant, which is not expected to last indefinitely. There is potential for bone and soft tissue remodeling which would result in maintenance of curve correction although this has not yet been established. When cords do break, it is usually after 2–3 years^38^, although this does not imply clinical failure, which is usually defined by residual major Cobb angle and indication for PSF^35,36,39^. For these patients, intraoperative correction, the ability to delay PSF, and the potential for curve correction with additional flexibility outweighed potential downsides, and ultimately aligned with their values. However, the purpose of our present study is to evaluate early surgical morbidity of the AVBT procedure which should not be impacted by skeletal maturity.

## Conclusions

AVBT has demonstrated some success, but as a novel procedure, there is still much to be learned. For select candidates, AVBT may be an appropriate treatment but decisionmakers should understand that the complications may be more pulmonary in nature than those seen in PSF. And while motion preservation is an important benefit of AVBT, its main goal is to maintain curve correction through growth modulation. Surgeons who perform AVBT should be cautious in their use of rib resection to optimize screw trajectory, as this was strongly associated with major complications.
